# Surgical Approach to Bilateral Impacted and Inverted Mesiodentes in a Nonsyndromic Pediatric Patient: A Case Report and Brief Literature Review

**DOI:** 10.7759/cureus.80926

**Published:** 2025-03-20

**Authors:** Nhung T Nguyen, Quang V Dang, Vinh Q Dang

**Affiliations:** 1 Department of Pediatric Dentistry and Orthodontics, Faculty of Odonto-Stomatology, Can Tho University of Medicine and Pharmacy, Can Tho, VNM

**Keywords:** bilateral, impacted, inverted, mesiodentes, supernumerary teeth

## Abstract

Supernumerary teeth (ST) are a form of abnormal dental development and may not always present with symptoms. This case report discusses an eight-year-old child who presented with spacing in the maxillary anterior region. Radiographic examination revealed bilateral impacted and inverted mesiodentes, which were successfully managed through surgical extraction and orthodontic treatment. The postoperative course was uneventful, resulting in satisfactory outcomes for both the patient and his parents. We emphasize the importance of periodic check-ups during the early stages of tooth exfoliation, including proactive clinical and radiographic evaluations for the early diagnosis and management of multiple ST. Along with a review of the literature, this report suggests that a timely surgical approach should be indicated to effectively address the patient’s condition, ensuring optimal outcomes with minimal intervention.

## Introduction

Supernumerary teeth (ST), also referred to as hyperdontia, is a developmental anomaly characterized by an excessive number of teeth in addition to normality. ST can be single or multiple, as well as symmetric or asymmetric in any region of the dental arch [1]. The most common type of ST is mesiodens, which emerges between the maxillary central incisors [2]. Several theories have been proposed to explain the development of ST, including atavism, dichotomy, and hyperactivity of the dental lamina [3]. In addition, various oral disorders and hereditary syndromes have been associated with its occurrence [4]. However, the precise pathological mechanisms of ST remain poorly understood [3].

ST-related complications may include dental impaction, ectopic eruption, delayed eruption, malocclusion, root dilaceration, and cyst formation [1]. ST may be retained if they do not cause any adverse effects or may be considered as replacement teeth if they fulfill a functional and aesthetic role in the absence of the relevant permanent teeth [5]. However, surgical extraction is frequently indicated to address these complications. Herein, we present a nonsyndromic case involving bilateral impacted and inverted mesiodentes, which was successfully treated with surgical extraction followed by orthodontic treatment. This report aims to contribute a typical case of bilateral impacted and inverted mesiodentes, accompanied by a brief literature review, emphasizing the importance of early treatment to address both the pathological and cosmetic aspects of the patient’s condition.

## Case presentation

**Figure 1:**
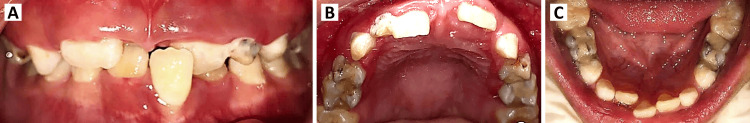
Intraoral photographs taken at the initial examination. (A) Frontal view. (B) Maxillary occlusal view. (C) Mandibular occlusal view

An eight-year-old Vietnamese boy accompanied by his mother to visit the Department of Odonto-Stomatology with a chief complaint of spacing in the maxillary anterior region. His medical and dental history revealed no associated systemic diseases or allergies. Intraoral examination displayed a very large diastema (approximately 9 mm) between permanent maxillary central incisors, a crossbite between the permanent maxillary left central incisor and the permanent mandibular left central incisor, as well as the presence of dental plaque, tartar, and caries (Figure 1).

**Figure 2:**
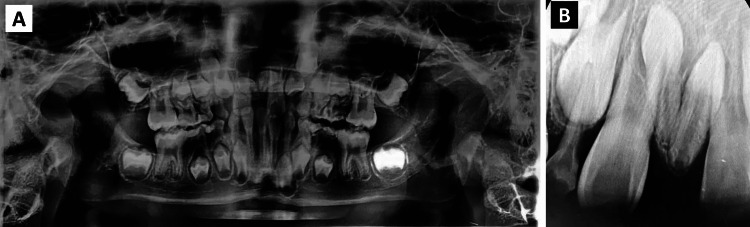
Preoperative radiographs revealing bilateral impacted and inverted mesiodentes. (A) Panoramic radiograph. (B) Periapical radiograph

Radiographic examination, including panoramic and periapical radiographs, revealed the presence of two impacted and inverted ST between the permanent maxillary central incisors, which resulted in a diastema. Only one of these teeth exhibited nearly complete root formation (Figure 2). The proposed comprehensive treatment plan included surgical extraction, followed by orthodontic intervention.

**Figure 3:**
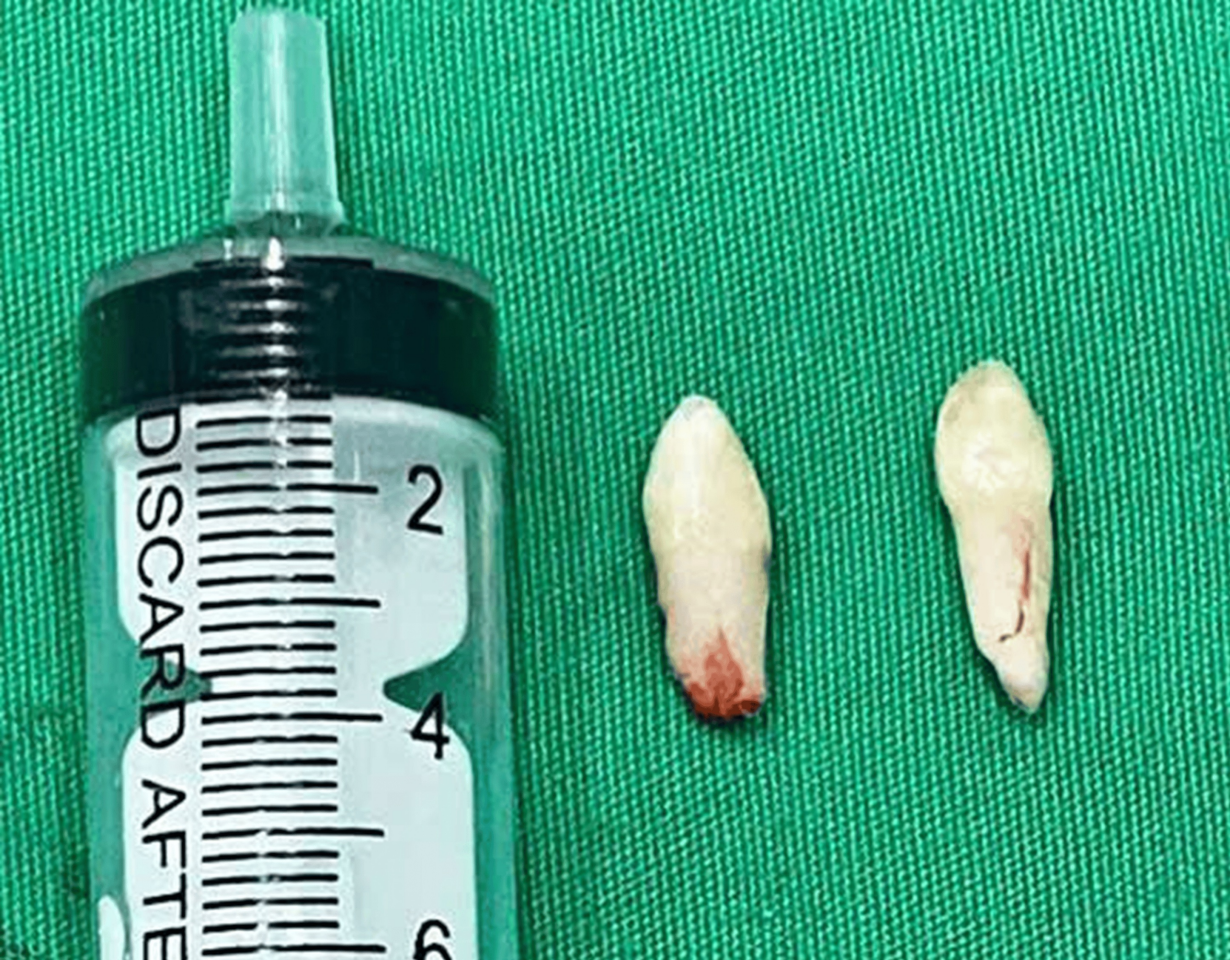
Bilateral mesiodentes extracted with the entire crowns and roots Syringe placed for scale

During the preoperative treatment, the child underwent scaling with an ultrasonic scaler. The surgical procedure was performed under local anesthesia and aseptic conditions. A three-sided flap was created using a periosteal elevator, a scalpel handle #3, and a scalpel blade #15. A low-speed handpiece with a No. 702 bur was utilized under abundant saline irrigation. The mesiodentes were removed by an elevator and forceps with the entire crowns and roots (Figure 3). The extraction socket was gently probed by a curette and flushed with saline to remove any surgical debris. The final step is the closure of the socket, where the flap was repositioned with a 3-0 nylon suture.

**Figure 4:**
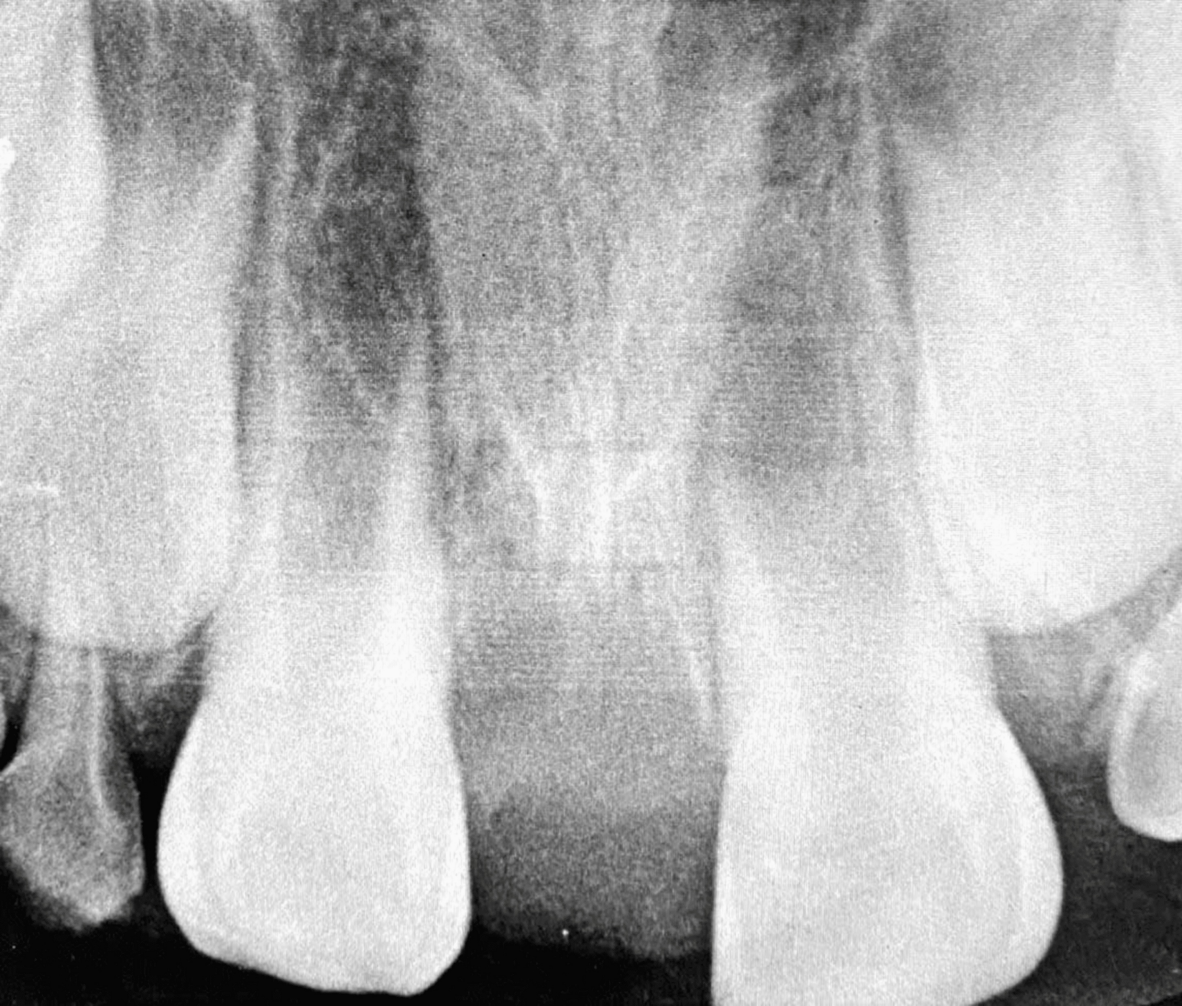
Periapical radiograph taken two weeks after surgery

**Figure 5:**
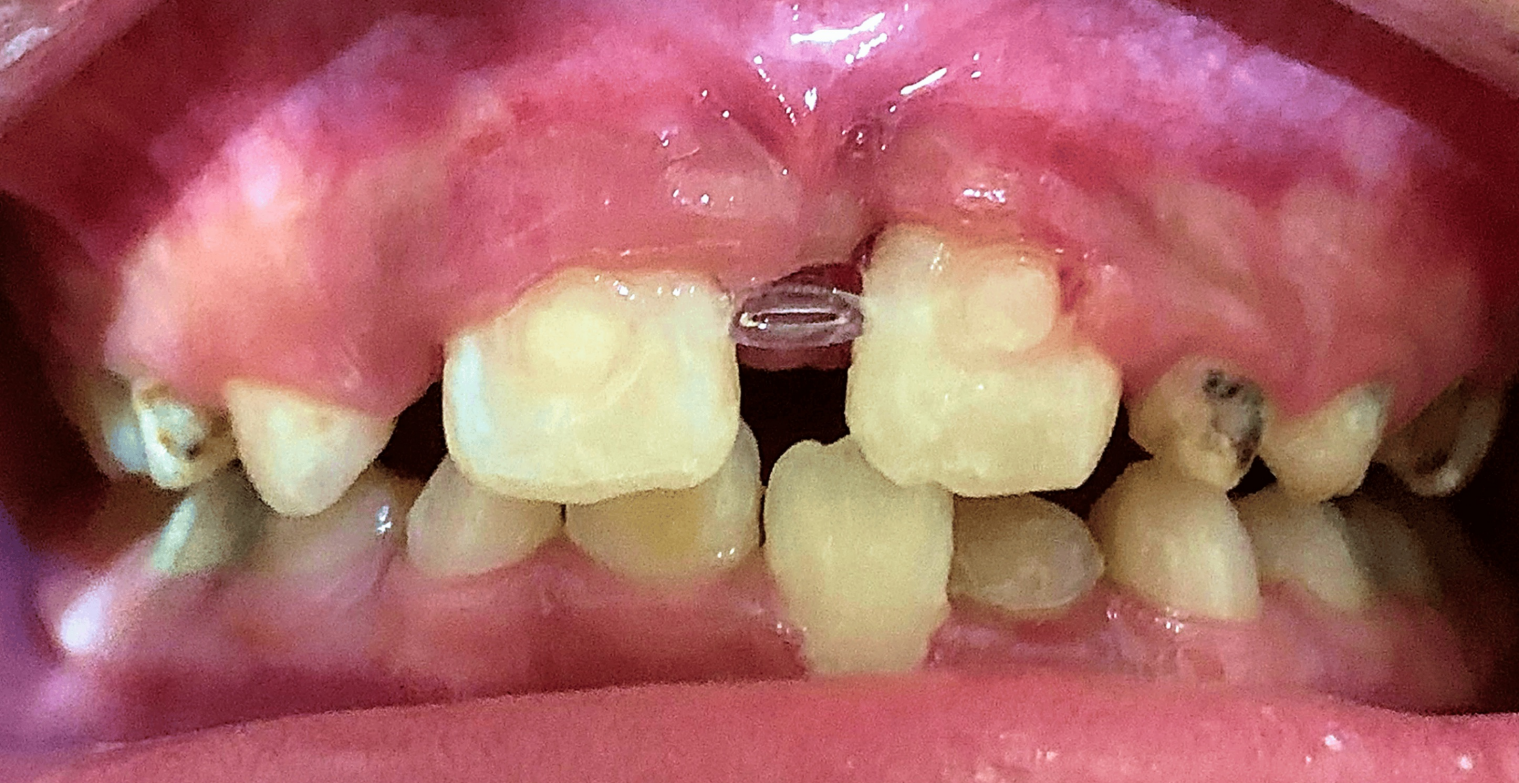
Intraoral photograph taken within one week of orthodontic intervention

The patient returned for suture removal, and a periapical radiograph was taken after two weeks, revealing good healing (Figure 4). The patient was then referred for orthodontic treatment to address the crossbite and diastema issues and continued to receive appropriate monitoring of the results over a six-month period (Figure 5).

## Discussion

The prevalence of ST ranges from 0.3% to 0.8% in deciduous dentition and from 1.5% to 3.5% in permanent dentition [1]. ST are more commonly found in the maxilla than the mandible and more frequently in men than women [4]. Among studies of the Asian population, ST are typically found as a single tooth in children with mixed dentition. Mesiodens, inverted direction, impacted status, and conical shape are the predominantly reported characteristics [2,6]. The literature review includes 13 reported cases of bilateral impacted and inverted mesiodentes in English to date, including the current case [7-16]. All of these cases were observed in Asian countries, with a male-to-female ratio of 10:3. More than half of the cases were in children aged 6-12 years, while the remaining cases involved older individuals, with the maximum age of 25. Most patients typically seek hospital or clinic care when complications arise (Table 1). Unlike other cases in the literature review, the case reported by Sharifi et al. was exceptional, as it involved the incidental recognition of bilateral impacted and inverted mesiodens during a routine dental check-up [14].

**Table 1:** Demographic characteristics and chief complaints in cases of bilateral impacted and inverted mesiodentes

Case	Study	Year	Country	Age	Sex	Chief complaint
1	Dinkar et al. [7]	2007	India	14 years	Female	Painful palatal swelling
2	Canoglua et al. [8]	2009	Türkiye	8 years	Male	Maxillary anterior crowding
3	Byatnal et al. [9]	2013	India	13 years	Male	Maxillary anterior swelling
4	Krishnappa et al. [10]	2014	India	16 years	Female	Maxillary anterior misalignment
5	Desai et al. [11]	2014	India	12 years	Male	Proclined maxillary anterior teeth
6				7 years	Male	Maxillary anterior teeth uneruption
7				25 years	Female	Discolored tooth on right maxilla
8	Viswanathan and Pai [12]	2015	India	13 years	Male	Palatal swelling and pain during swallowing
9	Al-Sehaibany et al. [13]	2016	Saudi Arabia	8.5 years	Male	Delayed eruption of maxillary anterior teeth
10	Sharifi et al. [14]	2021	Iran	9 years	Male	Routine dental checkup
11	Rajaram Mohan et al. [15]	2022	India	21 years	Male	Maxillary anterior absence
12	Koyama et al. [16]	2023	Japan	9 years	Male	Malocclusion
13	This case	2025	Vietnam	8 years	Male	Maxillary anterior spacing

Radiographic examination is a crucial and indispensable procedure for identifying ST regardless of the radiographic modality [17]. Table 2 presents the types of radiographic tools utilized in case reports from the literature review. While panoramic radiographs are commonly employed, they are not considered the most appropriate tool for identifying ST, and additional radiographs may be required for a precise diagnosis [18]. It is noteworthy that cone-beam computed tomography (CBCT) has recently been highlighted as the most effective application for ST diagnosis, offering accurate information on the number, shape, location, and proximity of vital neighboring anatomical structures [19]. In our department, although CBCT was not employed to identify the bilateral impacted and inverted mesiodentes due to the unavailability of advanced technical facilities, the combination of panoramic radiographs and periapical radiographs represents a cost-effective and practical diagnostic option for patient care.

**Table 2:** Clinical findings and treatment in cases of bilateral impacted and inverted mesiodentes Pano: panoramic radiograph; Occl: occlusal radiograph; PA: periapical radiograph; LC: lateral cephalogram; CBCT: cone-beam computed tomography; CT: computed tomography; SE: surgical extraction; OT: orthodontic treatment; N/A: not applicable

Case	Study	Year	Radiographic type	Clinical findings	Treatment	Follow-up
1	Dinkar et al. [7]	2007	Pano, Occl	Dentigerous cyst	SE	6 months
2	Canoglua et al. [8]	2009	Pano, Occl, PA, LC	Crowding	SE, OT	24 months
3	Byatnal et al. [9]	2013	CT	Dentigerous cyst	SE	N/A
4	Krishnappa et al. [10]	2014	Pano, Occl, PA, LC	Crowding	SE, OT	N/A
5	Desai et al. [11]	2014	Pano	Proclined anterior teeth	N/A	N/A
6			Occl	Proclined anterior teeth	N/A	N/A
7			Occl	Proclined anterior teeth	N/A	N/A
8	Viswanathan and Pai [12]	2015	Occl	Swelling on palatal vault	SE	12 months
9	Al-Sehaibany et al. [13]	2016	Pano, Occl, CBCT	Delayed central incisor eruption	SE, OT	12 months
10	Sharifi et al. [14]	2021	Pano, CBCT	Proximity to anterior nasal spine and tooth roots	SE	N/A
11	Rajaram Mohan et al. [15]	2022	Pano, CBCT	Promixity of anterior nasal spine	N/A	N/A
12	Koyama et al. [16]	2023	Pano, CT	Promixity of tooth root	SE/mixed reality	12 months
13	This case	2025	Pano, PA	Diastema	SE, OT	6 months

Among the ST-related complications observed in cases of bilateral impacted and inverted mesiodentes, crowding, diastema, delayed eruption, and proclination of adjacent permanent teeth were common, whereas two patients experienced significant issues with dentigerous cysts. Although the optimum timing for impacted ST extraction has been debated, some advocate for early diagnosis and intervention of impacted mesiodens to minimize future complications and the need for orthodontic treatment [19,20]. In terms of treatment, the unusual number, direction, and proximity to vital neighboring anatomical structures in these cases could increase the potential for exacerbated effects and necessitate specialized care if timely intervention is not provided. Nine out of 11 authors reported surgical extraction as the intervention method, while only two did not mention the type of procedure conducted in their patients. In the present case, the patient is in the ugly duckling stage, and a combination of surgical extraction and orthodontic treatment is considered the most appropriate option. We believe the surgical approach should be prioritized in cases of this condition due to the heightened risk involved.

Although the postsurgical period is also crucial to managing multiple ST, only about half of the reported cases included a monitoring duration of 6 to 24 months. In this case, the patient has been observed for six months, with a focus on bone healing progress, closure of the midline diastema, and proper eruption of remaining teeth, particularly the maxillary canines. The patient has not experienced any discomfort, and both he and his parents have expressed satisfaction with the positive outcome.

Despite the lack of advanced radiographic techniques, the current patient was successfully treated with a timely surgical approach and minimal orthodontic intervention, effectively avoiding significant ST-associated complications reported in other cases in the literature review. Nonetheless, we underscore that the utilization of CBCT should be strongly considered for comprehensive preoperative assessment in future research and clinical practice improvements, given its crucial role in evaluating the proximity to vital structures and aiding in surgical orientation. Furthermore, the literature review highlights that bilateral impacted and inverted mesiodentes may predominantly be observed only in Asian populations. However, this may not fully represent the global distribution of this condition due to the exclusion of case reports published in non-English languages or those with restricted access to full texts, which presents another limitation of our study.

## Conclusions

Most cases of bilateral impacted and inverted mesiodentes are not promptly identified until complications arise, including the present case. Therefore, surgical extraction should be regarded as the optimal treatment for addressing both aesthetic and pathological concerns in those individuals. Periodic check-ups, including comprehensive clinical and radiographic examinations, are essential for the early diagnosis and management of ST. Future research and clinical practitioners should recognize CBCT as a preferred radiographic modality for ST management, particularly in the preoperative evaluation of proximity to vital neighboring structures and surgical orientation.
